# Toward a synthetic hydrogen sensor in cyanobacteria: Functional production of an oxygen-tolerant regulatory hydrogenase in *Synechocystis* sp. PCC 6803

**DOI:** 10.3389/fmicb.2023.1122078

**Published:** 2023-03-22

**Authors:** Franz Opel, Marvin Amadeus Itzenhäuser, Isabel Wehner, Sara Lupacchini, Lars Lauterbach, Oliver Lenz, Stephan Klähn

**Affiliations:** ^1^Department of Solar Materials, Helmholtz Centre for Environmental Research – UFZ, Leipzig, Germany; ^2^Institute of Applied Microbiology (iAMB), RWTH Aachen University, Aachen, Germany; ^3^Institute of Chemistry, Technical University of Berlin, Berlin, Germany

**Keywords:** sensing and signaling, biotechnological hydrogen, regulatory hydrogenase, biosensor, synthetic biology, cyanobacteria

## Abstract

Cyanobacteria have raised great interest in biotechnology, e.g., for the sustainable production of molecular hydrogen (H_2_) using electrons from water oxidation. However, this is hampered by various constraints. For example, H_2_-producing enzymes compete with primary metabolism for electrons and are usually inhibited by molecular oxygen (O_2_). In addition, there are a number of other constraints, some of which are unknown, requiring unbiased screening and systematic engineering approaches to improve the H_2_ yield. Here, we introduced the regulatory [NiFe]-hydrogenase (RH) of *Cupriavidus necator* (formerly *Ralstonia eutropha*) H16 into the cyanobacterial model strain *Synechocystis* sp. PCC 6803. In its natural host, the RH serves as a molecular H_2_ sensor initiating a signal cascade to express hydrogenase-related genes when no additional energy source other than H_2_ is available. Unlike most hydrogenases, the *C. necator* enzymes are O_2_-tolerant, allowing their efficient utilization in an oxygenic phototroph. Similar to *C. necator*, the RH produced in *Synechocystis* showed distinct H_2_ oxidation activity, confirming that it can be properly matured and assembled under photoautotrophic, i.e., oxygen-evolving conditions. Although the functional H_2_-sensing cascade has not yet been established in *Synechocystis* yet, we utilized the associated two-component system consisting of a histidine kinase and a response regulator to drive and modulate the expression of a *superfolder gfp* gene in *Escherichia coli*. This demonstrates that all components of the H_2_-dependent signal cascade can be functionally implemented in heterologous hosts. Thus, this work provides the basis for the development of an intrinsic H_2_ biosensor within a cyanobacterial cell that could be used to probe the effects of random mutagenesis and systematically identify promising genetic configurations to enable continuous and high-yield production of H_2_*via* oxygenic photosynthesis.

## 1. Introduction

The anthropogenic emission of greenhouse gases like carbon dioxide (CO_2_) derived from the usage of fossil resources is regarded as the major driver of climate change. To tackle this issue, new approaches need to be supplied toward a CO_2_-neutral society and economy. Molecular hydrogen (H_2_) is generally believed to be an ideal candidate as a future energy carrier due to its high energy density and greenhouse gas emission-free usage. Industrially, H_2_ is, however, still mainly obtained *via* steam reforming of natural gases, therefore relying on fossil resources and leading to a considerable greenhouse gas footprint (Howarth and Jacobson, 2021).

Biotechnological H_2_ production using microorganisms as whole-cell biocatalysts offers the advantage of a sustainable process based on renewable resources. These biological H_2_ formation routes encompass anaerobic fermentation using organic compounds as electron donors in, e.g., chemotrophic *Clostridium* and *Enterobacter* species or phototrophic sulfur and non-sulfur bacteria, as well as oxygenic photosynthesis using algae and cyanobacteria (Mahidhara et al., 2019). Approaches based on oxygenic photosynthesis appear most promising as they rely on electrons that have been obtained from light-dependent oxidation of water. Cyanobacteria are the only prokaryotes capable of this process. Great effort has been made to optimize H_2_ production within cyanobacterial models such as the unicellular strain *Synechocystis* sp. PCC 6803 (hereafter referred to as *Synechocystis*). However, the breakthrough to enable continuous H_2_ production in whole-cell cyanobacterial catalysts has not been achieved yet. Currently, it suffers from low yields and rates as well as the prototypical molecular oxygen (O_2_) sensitivity of the enzymes involved in the formation of H_2_, namely hydrogenases or nitrogenases. Hydrogenases are metalloenzymes that perform the reversible splitting of H_2_ into protons and electrons. They are grouped based on the composition of their active site into nickel-iron [NiFe]-, iron–iron [FeFe]-, and iron [Fe]- or Hmd-hydrogenases (Lubitz et al., 2014). Previous studies tackled, for instance, the catalytic performance of H_2_ production by introducing highly active, heterologous [FeFe]-hydrogenases into *Synechocystis* (Berto et al., 2011; Wegelius et al., 2018) or by fusing the endogenous [NiFe]-hydrogenase to photosystem I for a direct electron transfer from photosynthesis (Appel et al., 2020). The O_2_ sensitivity has been addressed, for example, by introducing a heterologous O_2_-tolerant [NiFe]-hydrogenase (Lupacchini et al., 2021). Another strategy enabling a continuous hydrogenase activity would be the spatial separation from O_2_, e.g., through the encapsulation in synthetic microcompartments as demonstrated in *Escherichia coli* (hereafter referred to as *E. coli*) (Li et al., 2020). Moreover, metabolic engineering might target the redirecting of electron flows from competing pathways, like respiration and nitrate assimilation, to H_2_ evolution (Baebprasert et al., 2011). Nevertheless, further research and alternative approaches are required to overcome the known as well as yet unknown limitations and to make photosynthesis-driven H_2_ production amenable for biotechnological applications in the future (Bühler et al., 2021). In this regard, biosensors that respond to H_2_ in an easily detectable way could help to enable, e.g., a systematic screening of mutant libraries and the selection of those that are beneficial for H_2_ production.

Synthetic biosensors based on engineered bacterial cells, that respond to certain input stimuli with a desired output signal, can be designed by harnessing natural signal transduction systems to drive the expression of a reporter gene (Zhang et al., 2015; Ni et al., 2021). Also cyanobacteria have already been used as hosts to implement such cascades, e.g., for the intracellular sensing of heavy metals (Lacey et al., 2019; Patyi et al., 2021), O_2_ (Immethun et al., 2016), or toluene (Inaba et al., 2018). Natural H_2_-responsive systems were described in *Bradyrhizobium japonicum* (*B. japonicum*) (Black et al., 1994; van Soom et al., 1997, 1999), *Rhodobacter capsulatus* (*R. capsulatus*) (Dischert et al., 1999; Elsen et al., 2003), and *Cupriavidus necator* (also known as *Ralstonia eutropha*) H16 (hereafter referred to as *C. necator*) (Lenz et al., 1997; Lenz and Friedrich, 1998). The purple non-sulfur bacterium *R. capsulatus* has already been engineered to follow H_2_ production in co-cultivated green algae (Wecker et al., 2011). However, such a co-cultivation approach impedes the use as a tool for efficient mutant screening and is not feasible in the case of cyanobacteria as most bacterial strains do not grow in cyanobacterial growth media. In the long-term, a cyanobacterial biosensor strain that directly responds to intracellularly evolved H_2_ appears promising to use it as platform for a systematic optimization of H_2_ production within the same cell.

**Figure 1:**
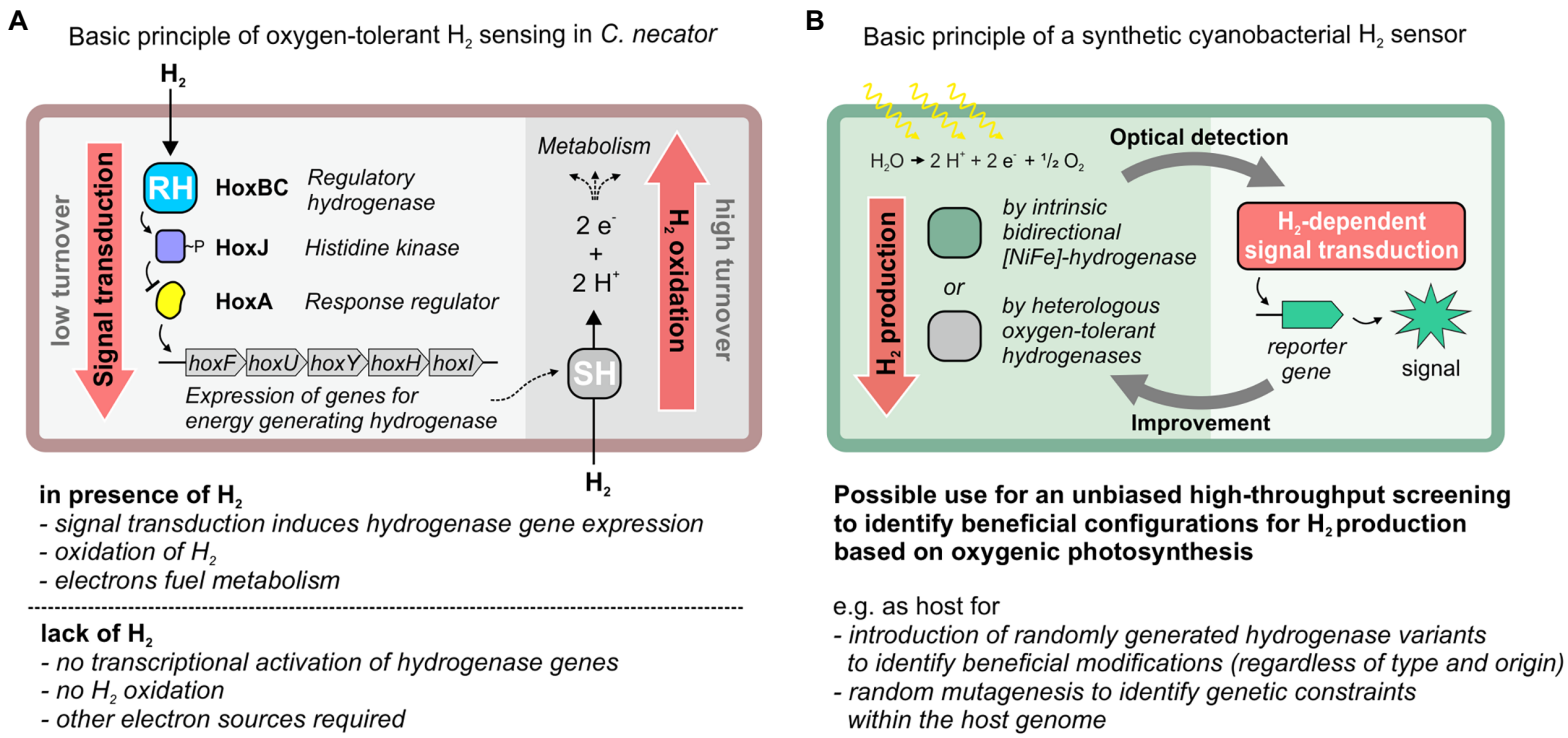
H_2_-dependent signal transduction in *C. necator* and its potential utilization for the design of a cyanobacterial H_2_ sensor strain that could be used as platform for the improvement of sustainable H_2_ production *via* oxygenic photosynthesis. **(A)** H_2_-responsive signal cascade in *C. necator* that enables the utilization of molecular hydrogen (H_2_) when other (favored) energy sources are absent. Signal transduction involves the regulatory hydrogenase (RH) module HoxBC as H_2_ sensor (with very low catalytic activity for H_2_ oxidation), the histidine kinase HoxJ and the response regulator HoxA, which promotes transcription of genes for two oxygen-tolerant hydrogenases [a soluble NAD^+^-reducing hydrogenase (SH) or a membrane-bound hydrogenase (MBH)]. These hydrogenases show high activity toward H_2_ oxidation and are used for energy-conservation. **(B)** Potential application of this H_2_-responsive signal cascade to drive reporter gene expression in cyanobacteria. This would enable optical detection of H_2_ that was produced by other hydrogenases using electrons obtained by light-driven water oxidation. This screening platform could facilitate strain engineering with diverse strategies, e.g., improving hydrogenase properties by (repeated) rational design or random mutagenesis, toward an improved photosynthetic H_2_ production. SH, soluble NAD^+^-reducing hydrogenase; RH, regulatory hydrogenase.

*Cupriavidus necator* has become the model organism for H_2_ oxidation in presence of O_2_. As a true “*Knallgas*” bacterium it can utilize H_2_ as sole electron donor and O_2_ as terminal electron acceptor. For this purpose, it uses O_2_-tolerant [NiFe]-hydrogenases. *C. necator* contains even four [NiFe]-hydrogenases that are O_2_-tolerant, among them the soluble NAD^+^-reducing (SH) and membrane-bound hydrogenase (MBH) as well as the regulatory hydrogenase (RH) (Lenz et al., 2015). The RH is a cytoplasmic enzyme and has a comparably simple structure composed of the two hydrogenase subunits, HoxB and HoxC (Pierik et al., 1998; Kleihues et al., 2000; Bernhard et al., 2001). The catalytic Ni-Fe center is coordinated by four cysteines to the protein matrix of the HoxC subunit (Winter et al., 2004). The active site iron carries three diatomic ligands, two cyanides (CN^−^) and one carbon monoxide (CO) (Pierik et al., 1998). Incorporation of the active site into the RH requires a set of seven auxiliary maturases, which are encoded by the *hypA1B1F1CDEX* genes (Buhrke et al., 2001; Bürstel et al., 2016). The RH serves as H_2_ sensor in combination with a two-component regulatory system and transmits the H_2_ input signal *via* the histidine kinase HoxJ to the response regulator HoxA, which functions as transcriptional activator for hydrogenase gene expression (Zimmer et al., 1995; Lenz et al., 1997; Schwartz et al., 1998; Figure 1A). Unlike canonical two-component systems, transcriptional activation is mediated by the unphosphorylated form of HoxA, and HoxJ phosphorylates/inactivates HoxA in the absence of H_2_ (Lenz and Friedrich, 1998). Notably, the phosphorylating activity of HoxJ is knocked out in its parental stain *C. necator* H16. To restore the native activity of HoxJ, a specific amino acid exchange is required, resulting in a functional HoxJ(S422G), also referred to as HoxJ* (Lenz and Friedrich, 1998). HoxJ* and the RH form the ternary H_2_-sensing complex (Buhrke et al., 2004). Production of active RH has already been established in *E. coli* (Lenz et al., 2007), very recently even under aerobic conditions (Fan et al., 2022).

In this study, we introduced the H_2_-sensing module of the *C. necator* RH, i.e., HoxB and HoxC, into *Synechocystis*. For this purpose, synthetic operons for the structural and accessory genes were designed for expression in *Synechocystis.* Heterologously produced and catalytically active RH was extracted from photoautotrophically grown cells that continuously evolve O_2_. Furthermore, as proof of concept, we introduced functional HoxJ* and HoxA into *E. coli* to modulate the expression of a reporter gene fused to a HoxA-responsive heterologous promoter. Our study provides the basis for further engineering of a cyanobacterial H_2_ biosensor strain that might enable a systematic screening of genetic setups and the selection of those beneficial for H_2_ production by the host-specific or other introduced hydrogenases (Figure 1B).

## 2. Materials and methods

### 2.1. Strains and culture conditions

*Escherichia coli* strains DH5α or JM109 were grown at 37°C either on agar-solidified LB medium or in LB liquid medium supplemented with 5 g L^−1^ NaCl under continuous shaking at 200 rpm. To select for the presence of certain plasmids the medium was supplemented with 100 μg mL^−1^ ampicillin, 35 μg mL^−1^ chloramphenicol, or 50 μg mL^−1^ spectinomycin. *C. necator* (obtained from the German Collection of Microorganisms and Cell Cultures, DSMZ) was grown at 37°C in LB liquid medium supplemented with 2.5 g L^−1^ NaCl under continuous shaking at 200 rpm. *Synechocystis* was cultivated in yBG11 (Shcolnick et al., 2007) liquid medium under continuous shaking at 150 rpm, or BG11 (Stanier et al., 1979) solidified with 1.5% (w/v) Bacto agar (Becton Dickinson) and supplemented with 3 g L^−1^ Na_2_S_2_O_3_. The cyanobacterial growth media were buffered with 10–50 mM HEPES to pH 7.2. Photoautotrophic growth conditions were set to 30°C, ambient CO_2_, constant light illumination with 50 μmol photons m^−2^ s^−1^, and 75% (v/v) humidity. For the selection of mutants, the media were supplemented with 10 μg mL^−1^ chloramphenicol, 20 μg mL^−1^ spectinomycin, or 50 μg mL^−1^ kanamycin. A non-motile, glucose tolerant strain of *Synechocystis*, originally received from Martin Hagemann (Rostock University, Germany), was used as the wild type (WT). The mutant *Synechocystis(Δhox)* that is devoid of the endogenous [NiFe]-hydrogenase was obtained from Kirsten Gutekunst (Kassel University, Germany). In particular, *hoxEFUYH* (*sll1220*-*sll1226*) have been replaced by a kanamycin resistance cassette (Appel et al., 2020).

### 2.2. Construction of plasmids and recombinant strains

*In silico* work was performed using the software Geneious (Biomatters). Genetic constructs were generated through standard molecular cloning procedures and maintained on plasmids in *E. coli* DH5α. DNA processing and recombination were performed using FastDigest restriction endonucleases (Thermo Scientific), T4 DNA ligase (Thermo Scientific), and FastAP thermosensitive alkaline phosphatase (Thermo Scientific) following the manufacturer’s instructions. The obtained constructs were verified by Sanger sequencing. Information about used oligonucleotides and plasmids is given in Supplementary Tables S1, S2.

**Figure 2:**
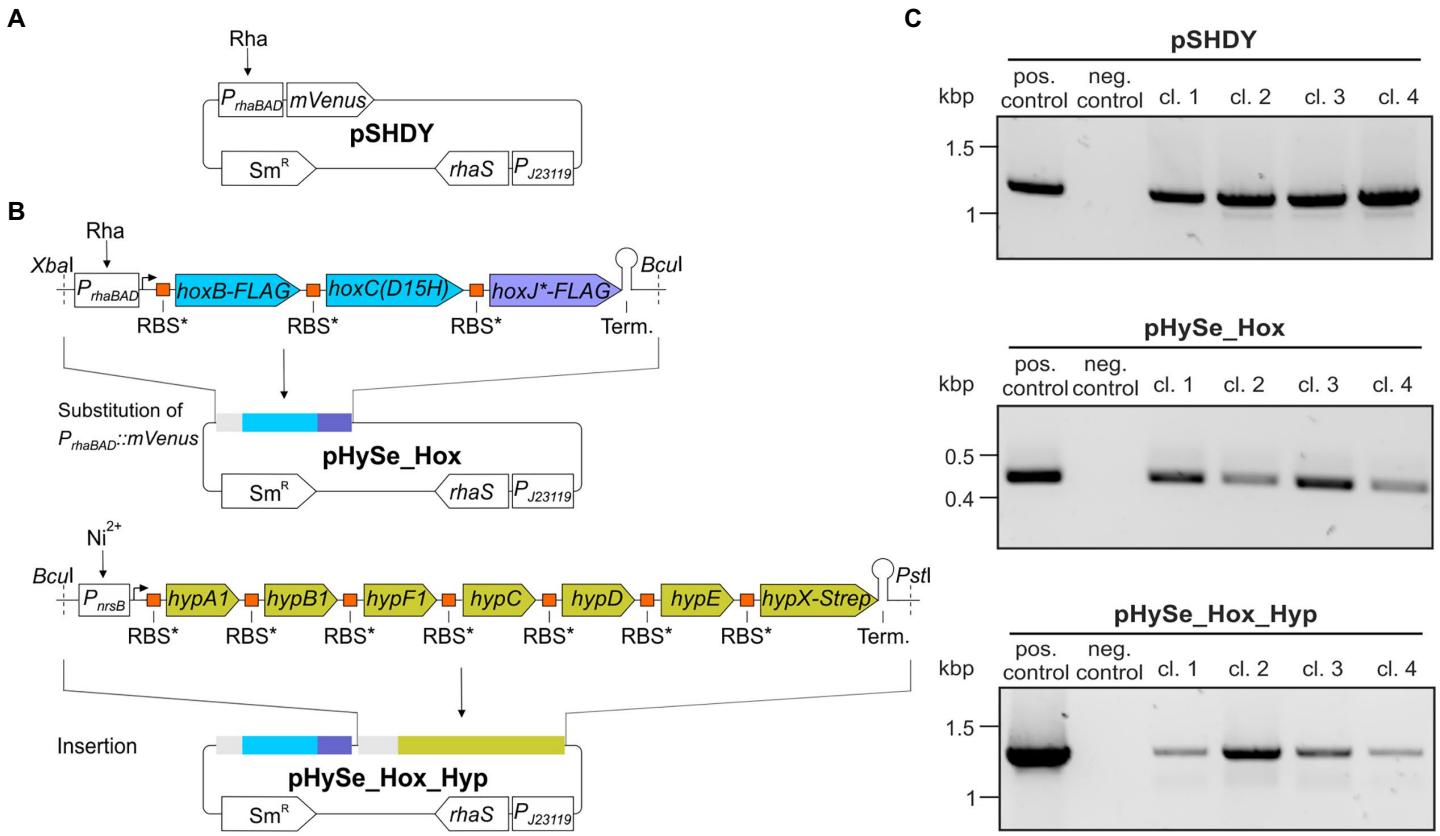
Scheme of synthetic operons for the expression of *C. necator* genes encoding the H_2_-sensing HoxBCJ* complex and its associated maturases in *Synechocystis*. **(A)** Structure of the plasmid pSHDY_*P_rhaBAD_::mVenus*_*P_J23119_*::*rhaS* (herein referred to as pSHDY). It can be maintained in *Synechocystis*, contains a spectinomycin selection marker (Sm^R^) for selection and enables L-rhamnose (Rha)-inducible reporter gene expression *via P_rhaBAD_* by featuring a cassette for the constitutive expression of *rhaS* (Behle et al., 2020). **(B)** Structure of the pHySe plasmids. The *hox* and *hyp* genes are organized in synthetic operons with a transcription terminator (Term.) at the 3′ ends. Their polycistronic transcription is driven by *P_rhaBAD_* and the nickel-ion (Ni^2+^)-responsive *P_nrsB_* (Englund et al., 2016), respectively. Each gene is interconnected by a ribosome binding site, RBS* (Heidorn et al., 2011). **(C)** Verification of recombinant *Synechocystis* strains. A *Synechocystis* parental strain lacking the endogenous hydrogenase was transformed using either pSHDY, pHySe_Hox, or pHySe_Hox_Hyp. Four selected clones (cl.1–4) were analyzed by colony PCR with primers that specifically targeted regions of the particular vector, i.e., S17 and S31 for pSHDY, S22 and S31 for pHySe_Hox, as well as S30 and S31 for pHySe_Hox_Hyp. The respective pure plasmid DNA served as positive control (pos. control). A PCR reaction lacking a template was used as negative control (neg. control).

The sequences for the design of synthetic operons encoding the H_2_-sensing complex and the corresponding maturases were obtained from the megaplasmid pHG1 of *C. necator* (Schwartz et al., 2003). The *hoxJ* sequence was modified to code for a variant exhibiting an amino acid substitution from serine to glycine at position 422, denoted as HoxJ* (Lenz and Friedrich, 1998). The *hoxC* sequence was altered to instead encode the variant HoxC(D15H) (Gebler et al., 2007). The gene sequences were codon-usage optimized for *Synechocystis* using the web-based tool JCat (Grote et al., 2005). The inducible promoters *P_rhaBAD_* (Behle et al., 2020) and *P_nrsB_* (Englund et al., 2016) were fused to the *hox* and *hyp* operons, respectively. The constructs were also equipped with unique restriction endonuclease sites flanking each operon, effective ribosome binding sites (RBS*, Heidorn et al., 2011) upstream of each ORF, as well as standardized transcription terminators (BioBrick BBa_B0015, Registry of Standard Biological Parts, 2003). Tag-encoding sequences were added to the 3′ ends of the open reading frames of *hoxB* (3xFLAG-tag), hoxJ* (3xFLAG-tag) and *hypX* (Strep-tag), respectively. The synthetic operons were chemically synthesized (Eurofins Genomics) and provided on plasmids, denoted as pHox2 and pHyp. For the maintenance in *Synechocystis*, the synthetic operons were transferred, either separately or combined, into the plasmid pSHDY_*P_rhaBAD_::mVenus*_*P_J23119_::rhaS* (Behle et al., 2020) *via Xba*I/*Bcu*I and *Bcu*I/*Pst*I sites, respectively. Thereby, the *P_rhaBAD_::mVenus* cassette was replaced. The resulting plasmids were named pHySe_Hox and pHySe_Hox_Hyp (Figure 2). The full sequences including annotations are provided in the Supplementary Data.

Plasmids of the pFO series and derivatives of pSB1A2_*P_trc1O_* (Huang et al., 2010), all of which harbor various combinations of expression cassettes for the genes *hoxA*, hoxJ* and *sfgfp*, were generated *via* the Gibson assembly procedure (Gibson et al., 2009). For this, respective sequences were amplified *via* PCR using primers with 5′ extensions to create homologous overhangs for the desired assembly with other DNA fragments. The *P_SH_* promoter, including the 5′ untranslated upstream region of *hoxF* (Zimmer et al., 1995; Schwartz et al., 1998), was amplified from genomic DNA of *C. necator* using primer pair P17/P18. Together with the *sfgfp* reporter gene and a downstream BioBrick BBa_B0015 transcription terminator, which were generated through PCR with primers P16/Sam_102 from pSEVA351-*sfgfp* (Opel et al., 2022), it was used for the assembly of pFO6 utilizing *Kpn*I-treated pSEVA351 (Martínez-García et al., 2020) as vector. The *hoxA* gene was amplified from gDNA of *C. necator* using P25 that additionally contained the ribosome binding site BioBrick BBa_B0034 (Registry of Standard Biological Parts, 2003) as 5′ extension and P26 fusing a sequence encoding a hexahistidine-tag at the 3′ end of the gene. The linear fragment was used for the assembly with *Bcu*I-cut pSB1A2, yielding pSB1A2_*P_trc1O_-hoxA*. pSB1A2_*P_trc1O_-hoxA^D55A^* has a substitution at the codon coding for the amino acid at position 55 of HoxA from 5′-GAT-3′ (Asp) to 5’-GCC-3′ (Ala). It was generated with the *Dpn*I-digested product from an inverted PCR, taking pSB1A2_*P_trc1O_-hoxA* as template and the primer pair P52/P53, as well as the homologous overhangs-supplying HoxA(D55A) double-stranded DNA fragment. The *P_trc1O_-hoxA* and *P_trc1O_-hoxA^D55A^* constructs were PCR-amplified using primers P49 and P50 from either pSB1A2_*P_trc1O_-hoxA* or pSB1A2_*P_trc1O_-hoxA^D55A^*, thereby fused to a 3’sequence encoding a Strep-tag II, instead of the His-tag, to be each inserted into *Bcu*I-linearized pSEVA351, yielding pFO25 and pFO26, respectively. Analogously, these two synthetic gene constructs were inserted into *Bcu*I-cut pFO6, which resulted in pFO27 and pFO28, respectively. To obtain pFO45 and pFO46, pHox2 was first subjected to an inverted PCR using P83 and P84, thereby deleting *hoxB^FLAG^* and *hoxC^D15H^* as well as the particular upstream situated RBS*. This was followed by AQUA cloning, creating pHox5 that encodes the P_rhaBAD_::hoxJ*^FLAG^ cassette. The latter was excised by restriction with *Xba*I and inserted into *Xba*I-linearized pFO27 and pFO28, yielding pFO45 and pFO46, respectively. Sequences of the pFO series are provided in the Supplementary Data.

*Synechocystis* WT as well as *Synechocystis(Δhox)* parental cells were made electro-competent and transformed *via* electroporation as described previously (Brandenburg et al., 2021). Plasmid-harboring strains were selected on BG11 agar plates containing appropriate antibiotics. Plasmid presence was verified by colony PCR using suitable primers and the GoTaq MasterMix (Promega) according to the manufacturer’s instructions. Recombinant *E. coli* JM109 strains were generated by electroporation of electro-competent cells *via* standard procedures.

### 2.3. RNA isolation and transcript analyses

For the isolation of RNA, *Synechocystis* cells were grown until reaching an OD_750_ ~ 0.8. The cultures were subsequently supplemented with final concentrations of 0.1% (w/v) L-rhamnose and 5 μM NiSO_4_. After 24 h, cells were harvested by rapid vacuum filtration applying sterilized polyether sulfone filters (pore size 0.8 μm, PALL). RNA isolation was performed as described previously (Bolay et al., 2022). The RNA samples were treated with RNase-free DNase I (Thermo Scientific) according to the manufacturer’s instructions. Afterwards, cDNA was generated by applying the high-capacity cDNA reverse transcription kit (Thermo Scientific) as given in the manufacturer’s instructions. A total of ~0.4 ng cDNA were used as template for quantitative PCR. Amplification of specific regions within either the *rnpB* gene or *hypX* were performed using the GoTaq MasterMix (Promega) according to the manufacturer’s instructions and primer pairs rnpB_114F/rnpB_226R and S30/P88, respectively (Supplementary Table S1).

### 2.4. Protein extraction and Western blots

*Synechocystis* cells were grown in presence of elevated CO_2_ concentration of 2% (v/v) to an OD_750_ of ~2. To induce expression of the *hox* and *hyp* genes the medium was supplemented with final concentrations (f.c.) of 0.2% (w/v) L-rhamnose and 5 μM NiSO_4_. In addition, 17 μM (f.c.) ferric ammonium citrate was added to foster hydrogenase maturation similar to previous reports (Lupacchini et al., 2021). Samples were collected by centrifugation after 24 and 48 h. Cells were resuspended in 750 μL TBS lysis buffer (100 mM Tris, 150 mM NaCl, 1 mM PMSF, pH 7.5) and transferred to 2 mL Precellys tubes (Bertin), together with a mixture of glass beads (Sartorius) of 0.09–0.15, 0.17–0.18, and 0.5 mm diameter. Cell disruption was performed using a Precellys Evolution homogenizer (Bertin) equipped with a Cryolys cooling system (Bertin) for 4 × 30 s at 10.000 rpm with 30 s interim breaks for cooling. The samples were subsequently separated in supernatant (soluble extract) and sediment (crude extract) by centrifugation and subjected to protein concentration determination using a Bradford dye reagent ready-to-use solution (Thermo Scientific) according to the manufacturer’s instructions. Cell suspensions of recombinant *E. coli* JM109 strains were analogously treated to obtain soluble protein extracts. Those cells were beforehand cultivated as described for GFP fluorescence determination, but using 0.5% (w/v) D-glucose instead of glycerol. Protein separation was performed *via* SDS-PAGE using a total amount of 20 μg protein for each sample. For immunodetection *via* Western blots the separated proteins were transferred to nitrocellulose membranes of 0.45 μm pore size (GVS), followed by hybridization with either a Strep-Tactin horse radish peroxidase (HRP) (IBA Lifesciences GmbH) or a monoclonal ANTI-FLAG M2-Peroxidase conjugate (Sigma-Aldrich) according to the manufacturer’s instructions. Chemiluminescence was detected by using the substrate solutions WesternSure PREMIUM Chemiluminescent (LI-COR) or WesterBright ECL (advansta) and the Fusion FX7 EDGE V0.7 imaging system (VILBER), following the manufacturer’s instructions.

### 2.5. Hydrogenase activity assays

Pre-cultivation of *Synechocystis* was performed as described above and expression of heterologous genes was induced by 0.1% (w/v) L-rhamnose and/or 2.5 μM NiSO_4_. After 48 h, cells were harvested and disrupted analogously but using an alternative lysis buffer (5% (v/v) glycerol, 50 mM KPO_4_, 1 mM PMSF, pH 8). A *Synechocystis* strain harboring the *hoxFUYHW* genes encoding the SH from *C. necator* (Lupacchini et al., 2021) served as control. Approximately 400 μg of soluble proteins were separated *via* native PAGE and subjected to in-gel staining as previously described (Lupacchini et al., 2021) with few modifications. These concerned the supplementation of 90 μM phenazine methosulfate, additionally to 800 μM NAD^+^ and 60 μM nitro blue tetrazolium (NBT) in an H_2_-saturated activity buffer (50 mM Tris, pH 8). Furthermore, the incubation time was increased from ~0.5 h to ~2.5 h. Hydrogenase activity, i.e., the release of electrons from H_2_ oxidation, is indicated *via* a step-wise reduction of the electron transfer mediators NAD^+^ and/or phenazine methosulfate and the colorimetric dye nitro blue tetrazolium, which finally results in a visible precipitation of formazan (Ponti et al., 1978). The gels were subsequently decolorized from the remaining loading dye in activity buffer overnight. Afterwards, presence of HoxB and HoxJ* proteins was confirmed by blotting the same polyacrylamide gel and hybridizing the membrane with antibodies against the attached 3xFLAG-tag as described above.

### 2.6. GFP fluorescence determination

Recombinant *Synechocystis* strains containing plasmids pFO25 (negative control), pFO6 (*P_SH_::sfgfp*), pFO27 (*P_SH_::sfgfp* + *P_trc1O_::hoxA*), or pFO28 (*P_SH_::sfgfp* + *P_trc1O_::hoxA^D55A^*) were analyzed *in vivo* regarding GFP fluorescence. The detection was performed as described previously (Opel et al., 2022). GFP fluorescence determination in *E. coli* JM109 was conducted for recombinant strains harboring the following plasmids: pSEVA351 (negative control), pFO27 (*P_SH_::sfgfp + P_trc1O_::hoxA*), pFO45 (P_SH_::sfgfp + P_trc1O_::hoxA + P_rhaBAD_::hoxJ*), and pFO46 (P_SH_::sfgfp + P_trc1O_:: hoxA^D55A^ + P_rhaBAD_::hoxJ*). Single colonies from selective LB agar plates were picked to inoculate liquid LB medium pre-cultures that were grown for ~18 h at 37°C. 1% (v/v) of these suspensions were taken to inoculate second pre-cultures using M9* medium, supplemented with 0.001% (w/v) thiamine, 2 mM MgSO_4_, 0.4% (v/v) glycerol, US* trace elements solution, and buffered to pH 7.2. The M9* pre-cultures were incubated for ~24 h at 37°C. A volume of 100 μL of these cell suspensions were added to 10 mL M9* medium in baffled shake flasks, and the cells were further cultivated at 30°C instead, due to the temperature sensitivity of HoxA (Zimmer et al., 1995). These main cultures were supplemented with 10 μM IPTG and/or 0.2% L-rhamnose after 8 h, followed by another 16 h of cultivation. For GFP fluorescence determination, samples were diluted to an OD_600_ of ~0.5 with TBS buffer (100 mM Tris, 150 mM NaCl, pH 7.5) in a final volume of 1,200 μL. Technical triplicates (each 200 μL) were transferred into an opaque black flat microtiter 96-well-plate (Nunc), followed by fluorescence measurements using an Infinite 200 PRO microplate reader (Tecan; gain: 123, integration time: 20 μs, excitation bandwidth: 9 nm, emission bandwidth: 20 nm, z-position: 2000 μm, 25 flashes) and excitation/emission wavelengths of 485 nm/520 nm, respectively. Furthermore, the same sample was used to measure the absorption at 600 nm in a transparent flat microtiter 96-well-plate (Nunc), also using the Infinite 200 PRO microplate reader (bandwidth: 9 nm, 25 flashes). The blank of the TBS buffer background was subtracted from the values detected for the cell suspensions. The fluorescence intensities were finally normalized by division of respective OD_600_ values.

## 3. Results

### 3.1. Design of customized operons for the expression of a H_2_-sensing complex in a cyanobacterial host

To functionally produce the RH of *C. necator* in *Synechocystis*, we rationally designed two operons *in silico* that were generated *via* chemical synthesis and inserted it into a vector that can be maintained in *Synechocystis* (Figures 2A,B). In case of the active site-containing HoxC subunit, we made use of the amino acid exchange variant HoxC(D15H), which, in contrast to the native protein, supports H_2_-dependent growth of *C. necator* at an O_2_ level of up to 10% (Gebler et al., 2007). The resulting gene cluster for the biosynthesis of the H_2_-sensing complex comprised *hoxB, hoxC^D15H^,* and hoxJ*. For proper maturation of the catalytic [NiFe] center we also utilized the corresponding accessory genes *hypA1B1F1CDEFX* (Buhrke et al., 2001; Bürstel et al., 2016). In *C. necator*, these genes are organized in an operon structure with partially overlapping open reading frames (Schwartz et al., 2003). To ensure their correct expression in the cyanobacterial target organism, we altered the spatial organization by linking each *hox* and *hyp* gene by an artificial spacer region, resulting in separate translational units (Figure 2B). Furthermore, the synthetic ribosome binding site RBS* which functions in *Synechocystis* (Heidorn et al., 2011), was introduced upstream of every single gene to enable efficient translation initiation. For facile detection of the proteins, sequences encoding either a 3xFLAG-tag or a Strep-tag were fused to *hoxB*, hoxJ*, and *hypX* (Figure 2B). The hoxJ* and *hypX* genes were chosen because they are the dorsal genes of each particular operon. Detection of both proteins is considered representative of upstream gene expression.

All protein-coding sequences were codon-usage optimized for translation in *Synechocystis*. To drive the polycistronic transcription of *hox* and *hyp* gene clusters, we used the L-rhamnose-inducible promoter *P_rhaBAD_* from *E. coli* (Behle et al., 2020) and the nickel ion (Ni^2+^)-dependent promoter *P_nrsB_* of *Synechocystis* (Englund et al., 2016), respectively. Thus, this setup permits a selective induction as well as a tight and tunable transcription of the synthetic gene constructs P_rhaBAD_::hoxB^FLAG^C^D15H^J*^FLAG^ (*hox* operon) and *P_nrsB_::hypA1B1F1CDEFX^Strep^* (*hyp* operon) in *Synechocystis.* Moreover, both operons were equipped with insulating transcription terminators at their 3′ end.

The constructs were inserted into the replicative vector pSHDY_*P_rhaBAD_::mVenus* _*P_J23119_*-*rhaS* (hereafter referred to as pSHDY), which encodes the heterologous transcriptional regulator RhaS that enables rhamnose-inducible gene expression in *Synechocystis* (Behle et al., 2020). The *P_rhaBAD_::mVenus* cassette present in this pSHDY construct (Figure 2A) was replaced with the synthetic *hox* operon resulting in the plasmid pHySe_Hox. Subsequently, the *hyp* operon was inserted downstream to obtain the plasmid pHySe_Hox_Hyp (Figure 2B). The resulting plasmids pHySe_Hox and pHySe_Hox_Hyp, as well as the precursor construct pSHDY, were used individually for the transformation of *Synechocystis*. For transformation, a strain devoid of the endogenous [NiFe]-hydrogenase, designated *Synechocystis(Δhox)* (Appel et al., 2020), was used to prevent subsequent cross-reactions with the RH activity. Plasmid presence was verified in all obtained clones (Figure 2C).

### 3.2. The genes encoding the H_2_-sensing complex are expressed in S*ynechocystis*

**Figure 3:**
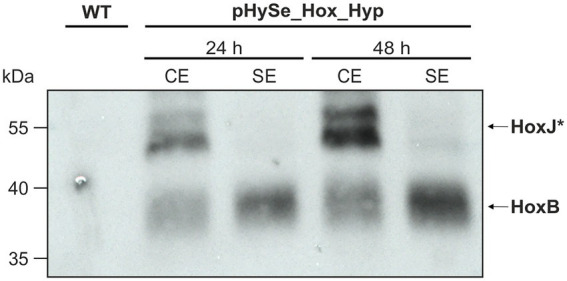
HoxJ* and HoxB from *C. necator* are synthesized in *Synechocystis*. Western blot for the detection of HoxJ*-FLAG and (~54 kDa) HoxB-FLAG (~40 kDa) fusion proteins. Samples were taken from *Synechocystis(Δhox)* that harbored the plasmid pHySe_Hox_Hyp, 24 and 48 h after induction with 0.2% (w/v) L-rhamnose and 5 μM NiSO_4_. Protein extract of a *Synechocystis* wild type (WT) strain served as negative control. CE, crude extract; SE, soluble extract.

C-terminal linkage with 3x-FLAG (HoxB & HoxJ*) or Strep-tags (HypX), enabled protein detection by commercially available antibodies targeting the corresponding tag. The *Synechocystis* strain harboring pHySe_Hox_Hyp was grown in the presence of L-rhamnose and Ni^2+^ to trigger *hox* and *hyp* gene expression, respectively (see section “Materials and methods” for specific inducer concentrations). To confirm heterologous gene expression, we performed immunoblotting to detect HoxB and HoxJ* using protein extracts of samples collected 24 and 48 h after induction. In addition to the crude extract, we also analyzed the soluble protein fraction obtained by centrifugation. In fact, distinct bands were detected, and their intensity increased according to the induction time. The bands represent HoxB-FLAG and HoxJ*-FLAG, as no signal was detected in the same size range in the *Synechocystis* wild-type strain (WT) (Figure 3). Consistent with the expected cytoplasmic localization of the RH, a strong signal for HoxB-FLAG was observed in the soluble extracts. However, the signal associated with HoxJ*-FLAG, which is also thought to be soluble, was predominantly present in the crude extract indicating partial protein misfolding or membrane association. Nevertheless, even though the signal was quite weak, a significant part was also found in the soluble fraction, in particular 48 h after induction. Thus, both fusion proteins were specifically detected, confirming the expression of the corresponding genes.

**Figure 4:**
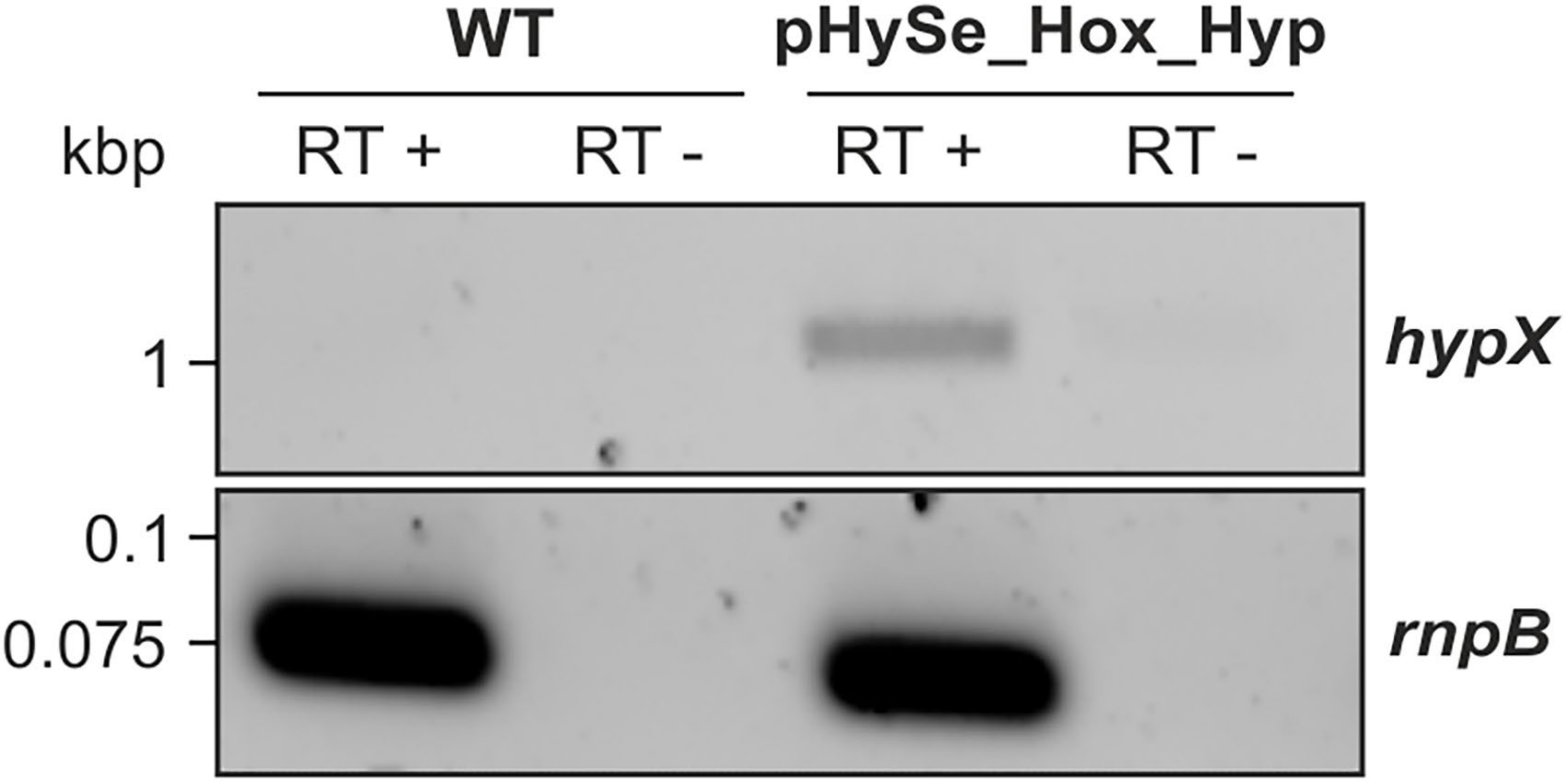
The polycistronic *hyp* mRNA is being transcribed in *Synechocystis*. PCR amplification of *hypX* or the housekeeping gene *rnpB* from cDNA that has been prepared from RNA isolates of *Synechocystis* wild type (control) or a strain harboring pHySe_Hox_Hyp. Samples in lanes denoted ‘RT +’ were treated with reverse transcriptase beforehand, while those indicated ‘RT −’ were not.

In case of HypX-Strep, however, no specific signal was observed in cells containing pHySe_Hox_Hyp (not shown), indicating either no translation or protein instability. To verify that the synthetic *hyp* operon is at least transcribed, we extracted total RNA from the same strain and performed classical reverse transcriptase (RT)-PCR targeting *hypX*. The reversely transcribed copy DNA (cDNA) for *hypX* was only detected in the *Synechocystis* strain containing pHySe_Hox_Hyp but not in the WT (Figure 4). That the band obtained is indeed a result of cDNA amplification of a *hypX* transcript was verified by a parallel RNA sample that was not treated with reverse transcriptase and consequently showed no bands for *hypX* and the housekeeping gene *rnpB*. As *hypX* is situated at the 3′ end of the synthetic gene cluster, we assume, that transcription of the upstream situated *hyp* genes also occurred. However, this analysis did not confirm the translation of the *hyp* gene transcripts. Nevertheless, sufficient synthesis of the maturation apparatus for the RH could be assumed, as indicated by the subsequent analysis (see below).

### 3.3. The H_2_-sensing complex is active when matured in *Synechocystis* cells growing photoautotrophically

**Figure 5:**
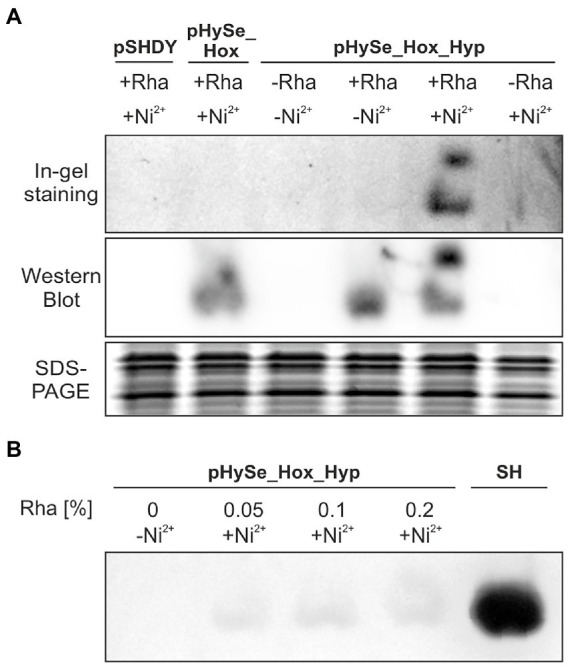
RH-mediated H_2_ oxidation in *Synechocystis*. **(A)** Recombinant *Synechocystis*(Δ*hox*) strains lacking the endogenous hydrogenase and containing either pSHDY, pHySe_Hox, or pHySe_Hox_Hyp were grown photoautotrophically under aerobic conditions in the presence of the inducers, i.e., 0.1% (w/v) L-rhamnose (Rha) and/or 2.5 μM Ni^2+^ (indicated by +/−). Soluble proteins were separated *via* native PAGE and subjected to in-gel H_2_-dependent formazan staining to localize the H_2_-sensing complex (upper panel), as well as immunological detection of the HoxB-FLAG and HoxJ*-FLAG proteins (middle panel). A denaturing SDS-PAGE was conducted as loading control (lower panel). **(B)** In-gel activity of cells incubated with the given inducers using different amounts of Rha in % (w/v). A *Synechocystis* strain recombinantly producing the highly active NAD^+^-reducing soluble hydrogenase from *C. necator* (SH) served as positive control.

To investigate if the recombinant gene expression indeed results in the formation of active H_2_-sensing RH, we analyzed the H_2_ oxidation activity in soluble extracts of photoautotrophically grown cells of *Synechocystis* strains harboring the plasmids pSHDY, pHySe_Hox, and pHySe_Hox_Hyp. To this end, we used an in-gel activity assay under an H_2_ atmosphere (Buhrke et al., 2004), which has also been used recently to confirm the activity of hydrogenases in *Synechocystis* (Lupacchini et al., 2021). The recombinant strains were cultivated in the presence of different inducer combinations to achieve independent expression of the hoxBCJ* genes (L-rhamnose) and the *hypA1B1F1CDEX* operon (Ni^2+^). Soluble protein extracts were prepared and subjected to native polyacrylamide gel electrophoresis. Strikingly, in-gel H_2_ oxidation activity was detected only for *Synechocystis*(pHySe_Hox_Hyp) induced with both L-rhamnose and Ni^2+^ (Figure 5A, In-gel staining panel). The resulting activity bands were located at the same positions as the bands in the immunoblot analysis (also based on the native gel), showing HoxB-FLAG in complex with HoxC as the hydrogenase core module, and potentially HoxJ*-FLAG (Figure 5A, Western Blot panel). As the production of both HoxB-FLAG and HoxJ*-FLAG has been confirmed by a previous Western blot (Figure 3), a separate detection of both proteins has not been performed in this case. No H_2_ oxidation activity was detected in the corresponding native gel, when the Hyp proteins required to produce catalytically active RH were absent, either due to the lack of the *hyp* genes (in case of strain pHySe_Hox) or the inducer Ni^2+^ (in case of strain pHySe_Hox_Hyp) (Figure 5A). Thus, expression of both the *hox* and *hyp* gene clusters is required to obtain detectable H_2_ oxidation activity for the H_2_-sensing RH in *Synechocystis*. While most [NiFe]-hydrogenases are inactivated by traces of O_2_ (Shafaat et al., 2013), the RH activity has shown to be O_2_-tolerant (Ash et al., 2015). Remarkably, the RH activity was observed in extracts of O_2_-evolving photoautotrophically grown *Synechocystis* cells, indicating that both the maturation process and the catalytic activity of the RH occurred in the presence of O_2_.

The RH activity in *Synechocystis* is comparatively low, as demonstrated by the long incubation time of ~2.5 h required to obtain detectable bands derived from H_2_-dependent NBT reduction in the activity gel. A *Synechocystis* control strain containing the highly active SH from *C. necator* (Lupacchini et al., 2021) showed significantly stronger signal intensities after ~0.5 h already. The low signal strength of the RH in *Synechocystis* could not be increased by enhanced L-rhamnose levels, suggesting a saturation at 0.05% (w/v) rhamnose and consequently no limitation of the RH structural proteins (Figure 5B). Altogether, these results demonstrate for the first time the functional production of a recombinant regulatory hydrogenase with low H_2_ turnover activity in a cyanobacterium.

### 3.4. Transcriptional regulation and its modulation by the associated two-component system in a heterologous host

**Figure 6:**
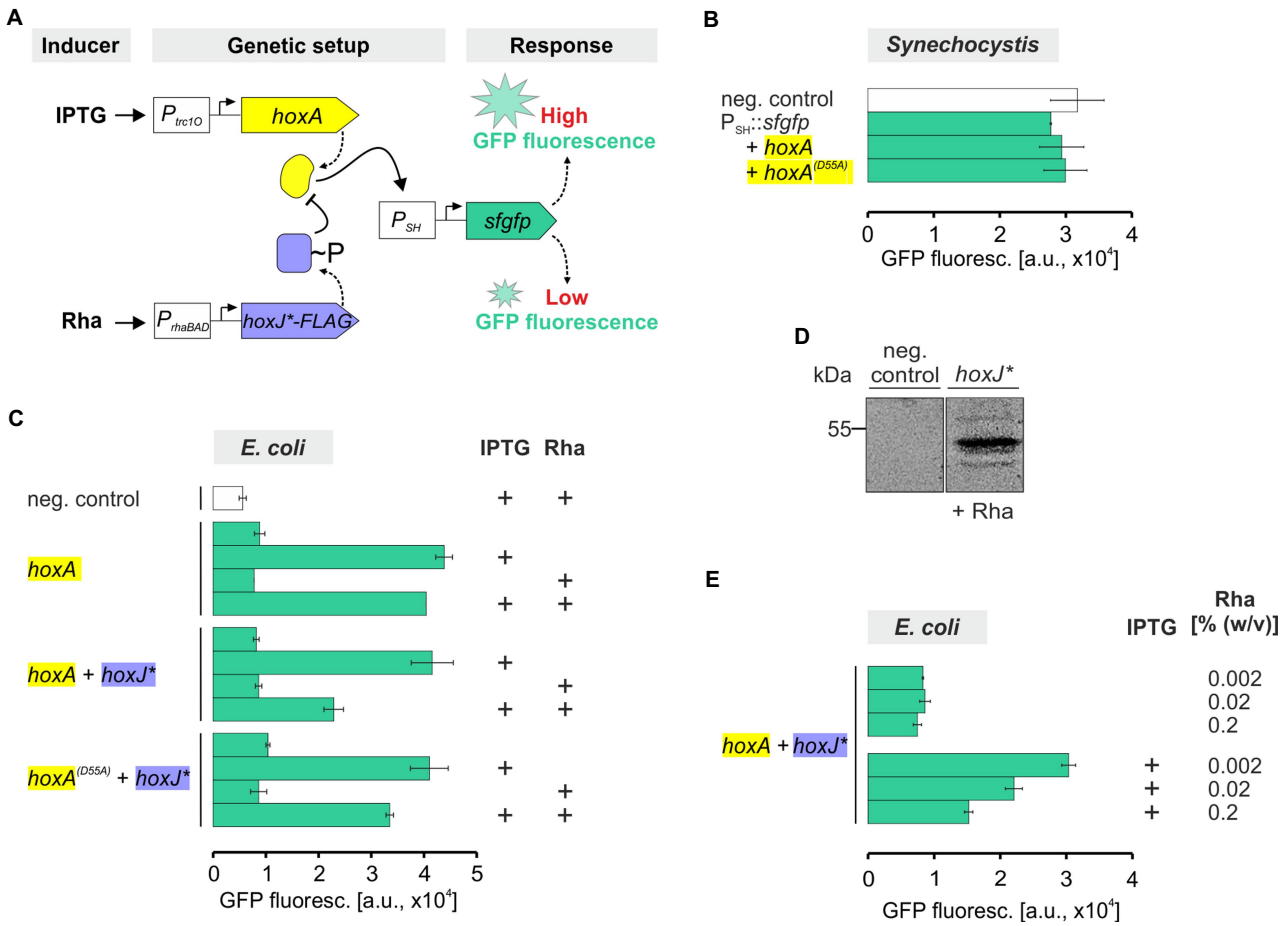
GFP fluorescence of *E. coli* and *Synechocystis* representing transcriptional regulation *via* HoxA and its modulation by HoxJ*. **(A)** Genetic setup of the generated strains. Expression of *hoxA* was driven by the IPTG-dependent *lac* promoter derivative *P_trc1O_* (Huang et al., 2010). The expression of the hoxJ* gene, fused to a FLAG-tag encoding sequence, was performed using the L-rhamnose (Rha)-inducible promoter *P_rhaBAD_* (Behle et al., 2020). HoxA-dependent expression of a reporter was achieved by fusion of a *sfgfp* gene to the cognate promoter of the NAD^+^-reducing, soluble hydrogenase gene cluster of *C. necator*, *P_SH_* (Zimmer et al., 1995; Schwartz et al., 1998). HoxJ* in turn modulates HoxA-dependent transcriptional regulation (Lenz and Friedrich, 1998). **(B,C,E)** Whole-cell GFP fluorescence of recombinant *Synechocystis* or *E. coli* JM109 strains harboring the synthetic *P_SH_::sfgfp* reporter gene construct in combination with different genetic information shown in panel **A**. Treatment with inducers IPTG and/or Rha prior to the measurements is indicated by ‘+.’ Detected fluorescence was normalized to OD_750_ (panel **B**) or OD_600_ (panels **C** and **E**) and is given in arbitrary units (a.u.). The particular negative controls (neg. control) contained a plasmid without reporter gene cassette. Data are the mean ± SD of at least two biological replicates (clones) each measured in technical triplicates. **(D)** Western blot confirming the presence of HoxJ*-FLAG (~54 kDa) in soluble protein extracts of *E. coli* JM109 harboring a P_rhaBAD_::hoxJ*^FLAG^ cassette versus an empty vector neg. control.

The final goal is to couple the functional RH with the associated kinase HoxJ* and the cognate response regulator HoxA to establish an H_2_-sensing signal transduction cascade in *Synechocystis*. In *C. necator*, HoxA positively controls the transcription of the genes encoding the SH and the MBH through binding to the promoters *P_SH_* and *P_MBH_*, respectively (Zimmer et al., 1995; Schwartz et al., 1998). The HoxA activity is modulated by HoxJ*-mediated phosphorylation, with transcriptional activation by HoxA in its non-phosphorylated state (Lenz and Friedrich, 1998). To drive HoxA-mediated gene expression in *Synechocystis*, the *hoxA* gene was set under control of the IPTG-inducible *P_trc1O_* promoter, which has been shown to provide sufficient constitutive expression due to the lack of the *lac* repressor LacI in the cyanobacterial host (Huang et al., 2010). Furthermore, a *sfgfp* reporter gene encoding the superfolder green fluorescent protein (Pédelacq et al., 2006) (hereafter referred to as GFP) was fused to the HoxA-dependent promoter *P_SH_* (Figure 6A). The synthetic gene constructs *P_SH_::sfgfp* and *P_trc1O_::hoxA* were introduced into *Synechocystis* WT, either separately or combined on a replicative plasmid. However, no significant GFP fluorescence beyond background activity was detected in any strain carrying the reporter gene construct alone or in combination with *P_trc1O_::hoxA* (Figure 6B). An inactivation of HoxA by unspecific phosphorylation could be excluded because the GFP fluorescence was similar in a reporter strain containing the phosphorylation-insensitive variant HoxA(D55A) instead of HoxA. This variant cannot be phosphorylated at the crucial aspartate at position 55 and has been shown to be always active (Lenz and Friedrich, 1998). Although several attempts have been made to improve *hoxA* expression, e.g., by using different promoters as well as codon-usage optimized gene variants, HoxA-dependent *sfgfp* expression has not yet been achieved in *Synechocystis* (not shown).

However, to validate the general functionality of our genetic constructs and to establish HoxA and HoxJ*-dependent gene expression in a heterologous system, we introduced the same replicative plasmids harboring *P_trc1O_::hoxA* and *P_SH_::sfgfp* into *E. coli* JM109. As this host contains LacI, *hoxA* expression is IPTG-inducible. Indeed, a ~ 5-fold higher GFP fluorescence was detected in cells grow in presence of IPTG compared to cells grown in absence of IPTG (Figure 6C). Under non-induced conditions, the GFP fluorescence was similar to the background autofluorescence of control cells harboring the empty vectors. Thus, in contrast to *Synechocystis*, the expected activity of HoxA to promote reporter gene expression *via* the *P_SH_* promoter was confirmed in *E. coli*.

Moreover, another synthetic gene construct encoding HoxJ* was included in the study to demonstrate the modulation of HoxA activity. HoxJ*-mediated phosphorylation of HoxA is expected to inactivate the response regulator (Lenz and Friedrich, 1998; Buhrke et al., 2004). A gene encoding HoxJ* carrying a C-terminal FLAG-tag (HoxJ*^FLAG^) was set under control of the L-rhamnose-inducible promoter *P_rhaBAD_*, and the resulting plasmid was introduced into the strain already harboring the Ptrc1O::*hoxA* construct. In general, this promoter could also be used in *Synechocystis* (Behle et al., 2020). Again, IPTG-induced *hoxA* gene expression resulted in GFP fluorescence. Remarkably, a significant decrease in GFP fluorescence was observed in cells that were grown in presence of both IPTG and L-rhamnose (Figure 6C). This correlates well with the exclusive detection of HoxJ* in protein extracts of cells grown in presence of L-rhamnose and carrying the respective gene construct (Figure 6D). The GFP level decreased to 50% compared to values obtained with strains either lacking the hoxJ* gene or that do not sufficiently express hoxJ* due to the absence of L-rhamnose. HoxA(D55A), which cannot be inactivated by HoxJ* through phosphorylation (Lenz and Friedrich, 1998), was again included as a control. As expected, a higher reporter signal was obtained with the corresponding strain in the presence of both IPTG an L-rhamnose than with the strain expressing wild-type *hoxA* as well as hoxJ* (Figure 6C). This result also suggests that the response regulator in *E. coli* JM109 is not inactivated by unspecific phosphorylation. Thus, for HoxA, an effective ~30% reduction in reporter signal strength was achieved by HoxJ* compared to HoxA(D55A), demonstrating the desired modulation. Moreover, the HoxJ*-mediated decrease of HoxA-dependent GFP fluorescence was further tunable by different amounts of L-rhamnose (Figure 6E).

## 4. Discussion

H_2_-based signal transduction cascades are considered being widespread, as the corresponding genes have been detected in many available genomes and metagenomes (Greening et al., 2016). Nevertheless, the biochemical and molecular mechanisms of H_2_ sensing have been studied in only a few representative bacteria to date. Ever since, these systems have been engineered and coupled with reporters. For example, *C. necator* has been engineered regarding a HoxBCJA-dependent expression of *lacZ* encoding β-galactosidase as reporter (Lenz and Friedrich, 1998). In principle, such recombinant strains could be utilized for the detection of H_2_ synthesized by other microbes, e.g., by co-cultivation or agar overlay assays on petridish basis (Wecker and Ghirardi, 2014). Similar to *C. necator,* the H_2_-sensing system of *R. capsulatus* consists of four proteins: HupUV which form the H_2_-sensing hydrogenase, the histidine kinase HupT, and the transcriptional regulator HupR that finally activates expression of an energy-generating uptake hydrogenase (Dischert et al., 1999; Elsen et al., 2003). This system has also been developed into a biosensor to screen large libraries of H_2_-producing nitrogenase variants in *R. capsulatus* directly (Barahona et al., 2016). Moreover, the corresponding strain has also been used as whole-cell biosensor to track H_2_ production in co-cultivated green algae (Wecker et al., 2011). However, in the case of cyanobacteria, such a co-cultivation approach is not feasible since most bacterial strains do not grow in cyanobacterial growth media lacking an organic carbon source.

In order to monitor H_2_ evolution within cyanobacterial cells, however, a signal transduction cascade must be transferred to the corresponding strain. This would allow *in vivo* screening of H_2_ evolution and the optimization of strains carrying, e.g., alternative hydrogenases (Figure 1). For example, Wecker et al. used engineered *R. capsulatus* strains that monitor H_2_ by reporter fluorescence to track the activity of a recombinant H_2_-evolving hydrogenase from *Clostridium acetobutylicum*. Only low H_2_ production has been detected, but the system potentially enables further screening and hydrogenase evolution approaches (Wecker et al., 2017). We successfully implemented two parts of the four-part H_2_-responsive signal transduction cascade from *C. necator* in the cyanobacterium *Synechocystis*. This is considered as a first step toward a synthetic cyanobacterial H_2_ biosensor that could be used analogously to previous reports (Wecker et al., 2017). A potential application would be, for instance, to optimize the H_2_ evolution activities of heterologously produced O_2_-tolerant, energy-converting hydrogenases, one of which was successfully implemented in *Synechocystis* recently (Lupacchini et al., 2021).

According to the current model, H_2_-sensing requires continuous H_2_ activation, i.e., H_2_ binding, H_2_ cleavage, as well as the corresponding proton and electron transfer (Lenz et al., 2015). Protein–protein complex formation with HoxJ* is if course also required (Buhrke et al., 2004; Löscher et al., 2010). Thus, continuous H_2_ oxidation is a prerequisite for H_2_-sensing. While the H_2_ oxidation activity of RH itself has been shown to be O_2_ insensitive (Buhrke et al., 2005), the signal transduction process is sensitive to high O_2_ levels. In this study, we therefore used a variant of the RH large subunit with an amino acid exchange near the active site, HoxC(D15H) (Gebler et al., 2007). The turnover rate of the native RH is almost two orders of magnitude lower than that of energy-conserving standard [NiFe]-hydrogenases (Bernhard et al., 2001). The H_2_ oxidation activity of the HoxBC(D15H) variant is indeed another two orders of magnitude lower, which explains the weak signals observed in the in-gel activity assay (Figure 5). However, it mediated native-like H_2_ signal transduction *in vivo* and supported H_2_-dependent growth of *C. necator* even at 10% O_2_, where signal transduction by the native RH was shown to be impaired (Gebler et al., 2007). The active-site containing HoxC subunit of RH also lacks a C-terminal extension that is typical for standard [NiFe]-hydrogenases and is proteolytically cleaved after insertion of the catalytic center (Kleihues et al., 2000). The comparatively simple structure, the lack of need for proteolytic quality control, the low H_2_ consumption, and of course the O_2_-tolerant H_2_-sensing ability make the RH an ideal candidate for a synthetic cyanobacterial H_2_ biosensor.

Here, we demonstrated that the recombinant RH in *Synechocystis* is synthesized in a catalytically active form, as evidenced by the H_2_ oxidation activity detected in protein extracts. Moreover, the cell extracts were obtained from photoautotrophically grown, O_2_-evolving cells. This shows that both the biocatalyst RH and its maturation machinery function properly under aerobic conditions in *Synechocystis*. In addition to the structural *hox* genes, the co-expression of the accessory *hypA1B1F1CDEX* genes of *C. necator* was required to achieve active RH. *Synechocystis* contains six endogenous Hyp proteins, likewise denoted HypA-F, that are responsible for the maturation of the bidirectional [NiFe]-hydrogenase of this organism (Hoffmann et al., 2006). However, *Synechocystis* lacks a homolog of the *C. necator* HypX. Based on our results, we cannot conclude whether all Hyp components or rather a reduced set from *C. necator* are necessary to achieve H_2_ oxidation activity of the RH in *Synechocystis*. The *Synechocystis* Hyp proteins may at least partially compensate for the maturation of O_2_-tolerant [NiFe]-hydrogenases from *C. necator* in the absence of the corresponding heterologous assembly apparatus. Notably, the NAD^+^-reducing [NiFe]-hydrogenase from *C. necator* was functionally produced in *Synechocystis*, without co-expression of the associated *hyp* genes (Lupacchini et al., 2021). However, it has been suggested that the *C. necator* Hyp proteins may be required for full SH activity as they have an amino acid sequence identity with the *Synechocystis* homologs of only 50–67%. Furthermore, HypX is required for aerobic maturation of the [NiFe]-hydrogenases of *C. necator* (Bürstel et al., 2016; Schulz et al., 2020).

The transfer of signal-responsive components into heterologous hosts usually includes a promoter and the associated transcriptional regulator (Fernandez-López et al., 2015; Sonntag et al., 2020; Ni et al., 2021). Our objective was to transfer the H_2_-sensing module (HoxBC) and the associated two-component regulatory system (HoxJ* and HoxA) to a cyanobacterial species to establish an H_2_-dependent transcriptional response. This has not yet been achieved in *Synechocystis*, presumably due to the absence of the minor sigma factor σ^54^ in cyanobacteria (Riaz-Bradley, 2019), which is required for the transcriptional activation of *P_SH_* and *P_MBH_* in *C. necator* (Zimmer et al., 1995; Schwartz et al., 1998). In addition, the DNA-bending integration host factor (IHF) may participate in hydrogenase promotor activation in *C. necator* (Zimmer et al., 1995; Schwartz et al., 1998). Accordingly, the introduction of this heterologous sigma factor or promoter engineering in *Synechocystis* should be pursued, which was beyond the scope of this study. However, as a proof of principle, we introduced HoxA and HoxJ* into *E. coli* to demonstrate the transcriptional regulation of a *sfgfp* reporter gene fused to *P_SH_*. Specific HoxA-dependent GFP fluorescence was detected, likely related to the presence of σ^54^ in *E. coli* (Jones et al., 1994). Our data are consistent with previous findings on functional, HoxA-controlled expression of a reporter gene fused to *P_SH_* in *E. coli* (Schwartz et al., 1998). Moreover, as expected, the kinase activity of HoxJ* clearly modulated the HoxA-dependent GFP fluorescence in *E. coli*, leading to a decreased reporter signal. Overall, we now have all genetic elements in hand to eventually assemble a functional H_2_ biosensor with optical readout in a cyanobacterium. This could ultimately be used to monitor cyanobacterial H_2_ production, e.g., to enable evolutionary or high-throughput screening approaches to improve hydrogenase properties as well as to circumvent existing constraints.

## Data availability statement

The original contributions presented in the study are included in the article/Supplementary material, further inquiries can be directed to the corresponding author.

## Author contributions

SK designed the study. FO, MAI, and IW constructed the plasmids. FO and IW performed the gene expression and RH activity analyses in *Synechocystis*. FO performed the GFP reporter assays. SL contributed to RH activity determination and experimental expertise for the in-gel assays. LL and OL contributed methodology and know-how on O_2_-tolerant hydrogenases. FO and SK wrote the manuscript with contributions from all co-authors. All authors contributed to the article and approved the submitted version.

## Funding

This project was initiated by a grant of the Max-Buchner Foundation to SK (MBFSt-Kennziffer: 3714). We acknowledge the use of the facilities of the Centre for Biocatalysis (MiKat) at the Helmholtz Centre for Environmental Research, which is supported by European Regional Development Funds (EFRE, Europe funds Saxony). We also acknowledge the use of the facilities of H2Saxony. This project (Nr. 100361842) is financed from funds of the European Regional Development Fund (EFRE) and co-financed by means of taxation based on the budget adopted by the representatives of the Landtag of Saxony.

## Conflict of interest

The authors declare that the research was conducted in the absence of any commercial or financial relationships that could be construed as a potential conflict of interest.

## Publisher’s note

All claims expressed in this article are solely those of the authors and do not necessarily represent those of their affiliated organizations, or those of the publisher, the editors and the reviewers. Any product that may be evaluated in this article, or claim that may be made by its manufacturer, is not guaranteed or endorsed by the publisher.
