# Supplementary material for: Toward a synthetic hydrogen sensor in cyanobacteria: Functional production of an oxygen-tolerant regulatory hydrogenase in Synechocystis sp. PCC 6803
Source: Front Microbiol. 2023 Mar 22;14:1122078. doi: 10.3389/fmicb.2023.1122078 (PMC10073562; doi:10.3389/fmicb.2023.1122078)
Supplement: Supplementary file 2 [file Table_1.DOCX]

Supplementary Material

# Supplementary Figures and Tables

**Table S1: Oligonucleotide list with names and sequences of used PCR primers.** 5’ primer extensions creating homologous overhangs and in case for the addition of further sequences are given in small letters, whereas the consensus binding regions are written in capital letters.

| **Name** | **Sequence (5’-3’)** |
| --- | --- |
| P16 | ATGAGCAAAGGAGAAGAACTTTTC |
| P17 | ggccgcgcgaattcgagctcggtacGAGGCGCTGGCACAAG |
| P18 | actccagtgaaaagttcttctcctttgctcatGTTGTCTCCTCCTTACTAATGTTCG |
| P25 | tgagcggataacaatttcacacatactagagaaagaggagaaatactagATGTCTGACAAGCAGGCCAC |
| P26 | ctttcgttttatttgatgcctggtactagtatcaatgatgatgatgatgatgTTTCTCCTCCAAGCCAAAGC |
| P49 | gtcgtgactgggaaaaccctggcgaTTGACAATTAATCATCCGGCTC |
| P50 | tggatctatcaacaggagtccaagactctagtatcatttttcgaactgcgggtggctccaTTTCTCCTCCAAGCCAAAG |
| P52 | ACGGGGGTGGAATTTCTC |
| P53 | GCACAGGATCACCGAGAC |
| P83 | tgaatactagagtagtggaggttactagATGTCCTCCAAACGCACC |
| P84 | acatctagtaacctccactactctagtaTTCATTACGACCAGTCTAAAAAGC |
| P88 | TTATTTTTCGAACTGCGGGT |
| Sam_102 | tcgactctagaggatccccgggtacTATAAACGCAGAAAGGCCCA |
| S17 | CGTTCGGTCAAGGTTCTG |
| S22 | CCTACGGCATTGTGGAAC |
| S30 | CCAAGTGTTTCACGAAGC |
| S31 | GAAAAATTGCCTACTGAGCG |
| rnpB_114F | GCCACAGAAAAATACCGCCC |
| rnpB_226R | CACCTTTGCACCCTTACCCT |
| HoxA(D55A) | TGCTGCGCCAGCCGGTCTCGGTGATCCTGTGCGCCCAACGCATGCCAGGCCTCACGGGGGTGGAATTTCTCAAAGAGGTGCGCGA |

**Table S2: Plasmids generated or used in this study.** Abbreviations: ori = origin of replication, Amp^R^ = ampicillin resistance, Cm^R^ = chloramphenicol resistance, Sm^R^ = spectinomycin resistance, MCS = multiple cloning site, codon = protein-coding sequence(s) codon-usage optimized for *Synechocystis*, FLAG = sequence coding for C-terminal triple FLAG protein tag, His = sequence coding for C-terminal hexahistidine protein tag, Strep = sequence coding for C-terminal streptavidin protein tag (Strep tag II), *P_rhaBAD_* = L-rhamnose-inducible promoter from *E. coli* (Behle et al., 2020), *P_nrsb_* = nickel ion-inducible promoter from *Synechocystis* (Englund et al., 2016), *P_trc1O_* = synthetic IPTG-inducible promoter (Huang et al., 2010), *P_J23119_* = BioBrick BBa_J23119 synthetic promoter (*Registry of Standard Biological Parts*), RBS* = synthetic ribosome binding site (RBS) (Heidorn et al., 2011), BioBrick BBa_B0034 = synthetic RBS (*Registry of Standard Biological Parts*), *rhaS* = gene encoding RhaS for transcription activation of *P_rhaBAD_*. For further descriptions see **Material & Methods**.

| **Plasmid** | **Relevant features** | **Reference** |
| --- | --- | --- |
| pHox | pUC19 ori, Amp^R^, *P_rhaBAD_*, RBS*, *hoxB^codon,^ ^FLAG^hoxC^D15H^*^,^ *^codon, His^hoxJ* ^codon,^ ^FLAG^* | This study |
| pHox2 | pUC19 ori, Amp^R^, *P_rhaBAD_*, RBS*, *hoxB^codon,^ ^FLAG^hoxC^D15H^*^,^ *^codon^hoxJ* ^codon,^ ^FLAG^* | This study |
| pHox5 | pUC19 ori, Amp^R^, *P_rhaBAD_*, RBS*, *hoxJ* ^codon, FLAG^* | This study |
| pHyp | pUC19 ori, Amp^R^, *P_nrsB_*, RBS*, *hypA1B1F1CDEX^codon, Strep^* | This study |
| pSHDY_*P_rhaBAD_::mVenus* _*P_J23119_*-*rhaS*  (in this study denoted as pSHDY) | RSF1010 ori, Sm^R^; *P_J23119_::rhaS* | (Behle et al., 2020) |
| pHySe_Hox | pSHDY backbone,  *P_rhaBAD_**::hoxB^codon,^ ^FLAG^hoxC^D15H^*^,^ *^codon^hoxJ* ^codon,^ ^FLAG^* | This study |
| pHySe_Hox_Hyp | pHySe_Hox backbone,  *P_nrsB_::hypA1B1F1CDEX^codon, Strep^* | This study |
| pSB1A2_*P_trc1O_* | pUC19-derived pMB1 ori, Amp^R^, *P_trc1O_* | (Huang et al., 2010) |
| pSB1A2_*P_trc1O_-hoxA* | pSB1A2_*P_trc1O_* backbone, BioBrick BBa_B0034, *hoxA^His^* | This study |
| pSB1A2_*P_trc1O_-hoxA^D55A^* | pSB1A2_*P_trc1O_-hoxA* backbone, *hoxA^D55A,^ ^His^* | This study |
| pSEVA351 | RSF1010 ori, Cm^R^, MCS | (Martínez-García et al., 2020) |
| pFO6 | pSEVA351 backbone, *P_SH_::sfgfp* | This study |
| pFO25 | pSEVA351 backbone, *P_trc1O_::hoxA^Strep^* | This study |
| pFO26 | pSEVA351 backbone, *P_trc1O_::hoxA^D55A, Strep^* | This study |
| pFO27 | pFO6 backbone, *P_trc1O_::hoxA^Strep^* | This study |
| pFO28 | pFO6 backbone, *P_trc1O_::hoxA^D55A, Strep^* | This study |
| pFO45 | pFO27 backbone, *P_rhaBAD_::hoxJ* ^codon, FLAG^* | This study |
| pFO46 | pFO28 backbone, *P_rhaBAD_::hoxJ* ^codon, FLAG^* | This study |

REFERENCES

Behle, A., Saake, P., Germann, A. T., Dienst, D., and Axmann, I. M. (2020). Comparative Dose-Response Analysis of Inducible Promoters in Cyanobacteria. *ACS Synth Biol* 9, 843–855. doi: 10.1021/acssynbio.9b00505

Englund, E., Liang, F., and Lindberg, P. (2016). Evaluation of promoters and ribosome binding sites for biotechnological applications in the unicellular cyanobacterium *Synechocystis* sp. PCC 6803. *Sci Rep* 6, 36640. doi: 10.1038/srep36640

Heidorn, T., Camsund, D., Huang, H.-H., Lindberg, P., Oliveira, P., Stensjö, K., et al. (2011). Synthetic biology in cyanobacteria engineering and analyzing novel functions. *Meth Enzymol* 497, 539–579. doi: 10.1016/B978-0-12-385075-1.00024-x

Huang, H.-H., Camsund, D., Lindblad, P., and Heidorn, T. (2010). Design and characterization of molecular tools for a Synthetic Biology approach towards developing cyanobacterial biotechnology. *Nucleic Acids Res* 38, 2577–2593. doi: 10.1093/nar/gkq164

Martínez-García, E., Goñi-Moreno, A., Bartley, B., McLaughlin, J., Sánchez-Sampedro, L., Pascual del Pozo, H., et al. (2020). SEVA 3.0: an update of the Standard European Vector Architecture for enabling portability of genetic constructs among diverse bacterial hosts. *Nucleic Acids Res* 48, D1164-D1170. doi: 10.1093/nar/gkz1024

*Registry of Standard Biological Parts*, http://parts.igem.org
